# Effects of adjunctive brexpiprazole with selective serotonin reuptake inhibitor treatment on anxiety and sleep architecture in mice

**DOI:** 10.1093/ijnp/pyag007

**Published:** 2026-02-20

**Authors:** Junya Maruoka, Kohei Kozuka, Ryo Egami, Yusuke Kubo, Yusuke Kakumoto, Tetsuro Kikuchi, Kazuhiko Kume

**Affiliations:** Tokushima Research Center for Drug Discovery, Otsuka Pharmaceutical Co., Ltd., Tokushima, Tokushima, Japan; Department of Neuropharmacology, Graduate School of Pharmaceutical Sciences, Nagoya City University, Nagoya, Aichi, Japan; Department of Neuropharmacology, Graduate School of Pharmaceutical Sciences, Nagoya City University, Nagoya, Aichi, Japan; Department of Neuropharmacology, Graduate School of Pharmaceutical Sciences, Nagoya City University, Nagoya, Aichi, Japan; Tokushima Research Center for Drug Discovery, Otsuka Pharmaceutical Co., Ltd., Tokushima, Tokushima, Japan; Tokushima Research Center for Drug Discovery, Otsuka Pharmaceutical Co., Ltd., Tokushima, Tokushima, Japan; Tokushima Research Center for Drug Discovery, Otsuka Pharmaceutical Co., Ltd., Tokushima, Tokushima, Japan; Department of Neuropharmacology, Graduate School of Pharmaceutical Sciences, Nagoya City University, Nagoya, Aichi, Japan

**Keywords:** brexpiprazole, major depressive disorder, residual symptoms, anxiety, sleep disturbance

## Abstract

**Introduction:**

Major depressive disorder (MDD) is frequently accompanied by residual symptoms such as anxiety and sleep disturbances, even after adequate antidepressant treatment. Brexpiprazole, a serotonin–dopamine activity modulator, has shown efficacy as adjunctive therapy for MDD. However, its mechanistic contribution to anxiety and sleep regulation remains unclear. This study aimed to evaluate the combined effects of brexpiprazole and paroxetine, a selective serotonin reuptake inhibitor (SSRI), on anxiety-like behavior and sleep architecture in mice.

**Methods:**

We used male Crl:CD1 and C57BL/6J mice. Anxiety-like behavior was assessed using the marble-burying behavior (MBB) test using Crl:CD1 mice. We also evaluated locomotor activity (LA) monitored to exclude sedative effects. Sleep architecture was evaluated via cortical electroencephalography and electromyography, quantifying Wake, rapid eye movement (REM) sleep, and non-REM (NREM) sleep stages using C57BL/6J mice.

**Results:**

In MBB test, paroxetine reduced buried marbles without affecting LA, whereas brexpiprazole alone was ineffective. The combination of brexpiprazole (0.1 mg/kg) and paroxetine (0.75 mg/kg) significantly decreased buried marbles. Next, to explore the role of α_2C_ adrenoreceptor (AR) antagonism, JP-1302 (a selective α_2C_ AR antagonist) was also tested. Similarly, JP-1302 (30 mg/kg) combined with paroxetine (0.75 mg/kg) decreased buried marbles. In sleep architecture, brexpiprazole dose-dependently decreased Wake and increased NREM sleep, while paroxetine primarily reduced REM sleep. Combined administration decreased Wake and REM sleep and increased NREM sleep. The combination showed effects comparable to each drug administered alone.

**Conclusions:**

In this study, we demonstrated that the combination of brexpiprazole and paroxetine produced anxiolytic-like effects and altered sleep architecture in mice. Furthermore, the anxiolytic-like effect of the combination suggests that brexpiprazole’s α_2C_ AR antagonistic activity may be one of several plausible contributors. Adjunctive brexpiprazole may influence anxiety and sleep architecture, potentially contributing to the management of residual symptoms in MDD.

## Graphical Abstract

**Graphical Abstract:**
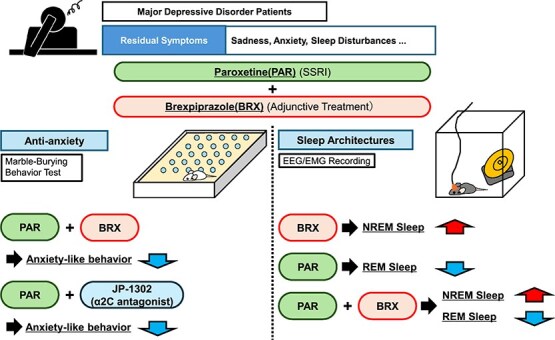

## Teaser

**Highlight**
 

- Brexpiprazole enhanced SSRI-related anxiolytic-like effects in mice.
- Brexpiprazole’s α_2C_ adrenoceptor antagonistic activity may partly contribute to anxiolytic-like effects in combination with paroxetine.
- Brexpiprazole and paroxetine showed distinct effects on sleep architecture.

**Significant outcomes**

The combination of brexpiprazole and paroxetine produced anti-anxiety effects at doses ineffective as monotherapy, suggesting enhanced effects in which brexpiprazole’s α_2C_ adrenoceptor antagonistic activity may be one of several plausible contributors. Regarding sleep architecture, brexpiprazole increased non-REM sleep while paroxetine reduced REM sleep, and their combination exerted effects comparable to each drug alone. These findings suggest that adjunctive brexpiprazole may help address residual symptoms such as anxiety and sleep disturbance in major depressive disorder, and further research focusing on these mechanisms could be crucial for improving treatment strategies.

**Limitations**

This study has several limitations. First, although we demonstrated anti-anxiety effects of both brexpiprazole and JP-1302 (an α_2C_ adrenoreceptor (AR) antagonist) in combination with paroxetine, the contribution of brexpiprazole’s α_2C_ AR antagonistic activity to these effects was inferred only indirectly. Second, given that mice are nocturnal, and the behavioral tests were conducted during the light phase, it is possible that the observed effects may not be reproduced in humans. Third, this study used only the marble-burying test to evaluate anxiety-like behavior. Because other behavioral tests (eg, elevated plus-maze test, open field test, zero maze test, conflict test) assess different aspects of anxiety, future studies using additional assays will help to clarify which types of anxiety symptoms are alleviated by the combination of brexpiprazole and paroxetine. Fourth, regarding the effects of brexpiprazole on sleep architecture, we primarily described the observed phenomena, and the involvement of specific receptors was hypothesized based on known Ki values, without direct mechanistic validation. Fifth, because brexpiprazole reduces locomotor activity at doses that increase NREM sleep, different doses were used for the marble burying test and the electroencephalography/electromyography experiments. Sixth, since only paroxetine was evaluated in this study, it remains unclear whether similar effects would be observed with other SSRIs or antidepressant classes. Finally, as the experiments were conducted in naïve mice. The study using disease model or genetically modified animals may provide insights that better predict clinical efficacy.

## INTRODUCTION

Major depressive disorder (MDD) is a highly prevalent disease characterized by various symptoms such as depressive mood, anxiety, anhedonia, decreased interest in pleasurable activities, poor concentration, sleep disturbance, or irritability.^1^ Major depressive disorder limits psychosocial function and diminishes quality of life.^2^ Selective serotonin reuptake inhibitors (SSRIs) are most often used and currently recommended as the first-line therapy for patients with MDD.^2–4^ However, residual depressive symptoms are common due to inadequate response to antidepressant treatment. In a clinical study, approximately one-third of patients achieved full remission, one-third experienced a partial response, and one-third were non-responders.^5^ In randomized controlled trials, although more than 90% of patients with MDD treated with the anti-depressant fluoxetine (an SSRI) met the criteria for remission, they still had at least one residual depressive symptom.^6^ The MDD patients with residual symptoms have a 3–6-fold higher risk of relapse.^5^ In open-label studies and randomized placebo-controlled clinical trials, it is reported that adjunctive therapy with second-generation antipsychotics had positive clinical effects on patients whose response to antidepressants was inadequate.^7^

Brexpiprazole is a serotonin–dopamine activity modulator and acts as a partial agonist at 5-HT_1A_ and dopamine D_2_ receptors, and an antagonist at 5-HT_2A_ receptors and α_1B/2C_ adrenoreceptors (AR).^8^ Brexpiprazole was developed as a treatment for schizophrenia and as an adjunctive treatment for MDD.^9–12^ Recently, brexpiprazole has been approved as a treatment for agitation associated with dementia due to Alzheimer’s disease.^13^^,^^14^

In preclinical studies, there have been many reports of the effects of brexpiprazole on depression and symptoms of anxiety. The combination of brexpiprazole and each of four drugs (escitalopram, fluoxetine, paroxetine, sertraline) reduced immobility time in the forced swim test (FST).^15^ For animal models of depression, social defeat stress and lipopolysaccharide-induced inflammatory stress increased the immobility time in both the tail suspension test (TST) and FST. Combination of brexpiprazole with fluoxetine improved the stress-induced change.^16^^,^^17^ Additionally, in a predator scent stress model, the combination of brexpiprazole and escitalopram improved anxiety-like behavior in the elevated plus maze test, and acute administration of brexpiprazole suppressed post-traumatic stress disorder (PTSD) like behavior.^18^^,^^19^ However, it is unclear what pharmacological activity of brexpiprazole plays a key role in these anti-anxiety effects.

Furthermore, it has been suggested that adjunctive treatment of brexpiprazole may improve sleep disturbances in MDD patients with inadequate response for antidepressant monotherapy. In fact, brexpiprazole improved not only the sleep efficiency score, total sleep time, sleep onset latency, wake-time after sleep onset, and the number of awakenings, but also improved the mean phase angle between peak cortisol and dim-light melatonin onset. These changes correlated with improvement of depressive symptoms.^20^^,^^21^ Sixty to 90% of depressed patients experience sleep disturbances, such as difficulties in initiating or maintaining sleep, and insomnia is also a risk factor for depression.^22–25^ Moreover, the most common residual symptoms were sleep disturbances (insomnia 48.2%, hypersomnia 35.9%).^6^ It has been reported that a combination of SSRIs and eszopiclone, a nonbenzodiazepine sedative hypnotic, enhances the ameliorative effects on depression.^26^ These reports suggest that treatment for sleep disturbance increases the antidepressant effects.

In this study, we evaluated the anti-anxiety effect of combination therapy with brexpiprazole and paroxetine (an SSRI) by using the marble-burying behavior (MBB) test. Additionally, we also evaluated its effect on sleep architecture by utilizing electroencephalography (EEG) and electromyography (EMG).

## MATERIAL AND METHODS

### Ethical Statements

This study is reported in accordance with the ARRIVE guidelines. All animal protocols were approved by Nagoya City University (approval number: NCU-YD21-001) and Otsuka Pharmaceutical Co., Ltd. (approval number: OPC-24-0286). All experiments in this study have been carried out in accordance with the Guide for the Care and Use of Laboratory Animals as adopted and promulgated by the U.S. National Institutes of Health, and all animals in this study were treated in accordance with the relevant Guidelines for Animal Care and Use at Otsuka Pharmaceutical Co., Ltd., or the Institutional Guidelines on Animal Experimentation of Nagoya City University.

### Animals

Male Crl:CD1 mice (23-33 g, 5 weeks old, Jackson Laboratory Japan, Inc., Japan) and male C57BL/6J mice (14-22 g, 5 weeks old, Clea Japan, Inc., Japan) were purchased. These strains were selected because they have been well validated in our laboratory for their respective experimental paradigms (behavioral tests and EEG/EMG recordings), and we have previously evaluated various compounds using these mouse strains, ensuring the reliability and reproducibility of the results. Mice were housed in a room maintained at 23 ± 2 °C with an alternating 12-h light–dark cycle. Food and water were available ad libitum.

### Drugs

In the MBB test and locomotor activity (LA) test, gum arabic (AG) was dissolved in distilled water to obtain a 5% solution (w/v). Brexpiprazole (Otsuka Pharmaceutical Co., Ltd.) and paroxetine hydrochloride (FUJIFILM Wako Pure Chemical Corporation) were dissolved in and diluted with the 5% AG solution. The brexpiprazole dose was selected based on pharmacokinetic data to approximate the anticipated exposure in clinically relevant adjunctive therapy. JP-1302 (Adooq Bioscience LLC.) was dissolved in and diluted with saline. In the EEG/EMG study, based on the previous study, we prepared a solution (vehicle) by dissolving 18% gelatin, 0.58% sucralose, and 0.28% sodium carboxymethylcellulose in dH_2_O in order to minimize the operational load on the recording device during administration.^27^ Brexpiprazole and paroxetine hydrochloride were dissolved in the vehicle. We gave each mouse 5% AG, saline, vehicle, or drugs at a volume of 10 mL/kg body weight.

### MBB Test

The anti-anxiety effect was evaluated during the light phase in relation to the number of buried marbles using a plastic cage (26 cm wide × 32 cm deep ×17 cm high) containing 25 glass marbles separated at equal intervals on 5-7 cm deep bedding. The lighting was adjusted to 500-600 lux at the center of the apparatus. After drug administration, each mouse was placed in the apparatus and allowed to move around freely. After 30 minutes, the mouse was removed from the apparatus and returned to its cage, and the observer counted the number of buried marbles. In this procedure, a marble is considered buried if 2/3 of the marble is covered with bedding. The observer was blinded to the treatment the mice had received.

### LA Test

After drug administration, each mouse was placed in a plastic box (25 cm wide × 25 cm high) with the bottom of the box covered in bedding material for 30 minutes. To mimic the conditions of the MBB test, we placed 5 marbles in the plastic box. Locomotor activity was automatically measured using an infrared ray passive sensor system (Supermex and CompACT AMS Ver.3; Muromachi Kikai Co., Ltd., Japan).

### Surgery (EEG/EMG Electrode Implantation)

Before the surgery, mice were habituated to a running wheel in the home cage (>3 days) and anesthetized by isoflurane and fixed using a stereotaxic apparatus (SM-6 M-HT, Narishige, Japan). Two of the three electrodes were placed on the cortex for EEG recording, while one was placed on the cerebellum as a ground. Two stainless steel screws for EEG were implanted at +1.0 mm AP and + 1.5 mm ML from bregma or + 1.5 mm AP and + 1.5 mm ML from lambda.^28^^,^^29^ The two stainless steel wires for EMG recording were inserted bilaterally into the trapezius muscles. The electrodes were fixed to the skull with dental cement. After surgery, mice were separately housed for a recovery period (>7 days) before recording.

### E‌EG/EMG Recording

The mouse was placed in an acrylic box (30 × 30 × 30 cm) equipped with a running wheel, with free access food and water. The mouse was habituated to this box. Next, EEG and EMG were obtained from mice able to move freely. EEG and EMG signals were obtained at a sampling rate of 100 Hz. These recorded data was processed with a bandpass filter in the range of 0-50 Hz and saved in digital format.

### Data Analysis for EEG/EMG Study

We analyzed the EEG and EMG signals with SleepSign software ver.3 (Kissei Comtec, Japan). We defined 1 epoch as 10 seconds, and each epoch was classified into Wake, rapid eye movement (REM) sleep, or non-REM (NREM) sleep stage based on the previous study.^30^ The waking stage was decided based on the EMG amplitude. Sleep stages were classified into REM sleep or NREM sleep according to the ratio of theta waves (4.0-10 Hz) or the density of delta waves (0.75-4.0 Hz), or EMG amplitude.^29^ We calculated the duration of waking, REM sleep, and NREM sleep per 1, 6, and 12 h for each mouse, and conducted frequency analysis for each stage by SleepSign. The EEG signals were analyzed using fast Fourier transform analysis from 0 to 20 Hz. The EEG spectral power in each frequency was normalized to the sum of that at 0-20 Hz in each Wake/REM/NREM stage. The NREM delta power was calculated as the AUC of the delta wave in the EEG spectral power.

### Statistics

All statistical analyses were performed using SAS Software for Windows, Release 9.4 (SAS Institute Japan). The sample size was determined based on a priori power analysis. GraphPad Prism 10.03 was used for graphical presentation. Results are presented as mean ± standard error of the mean (SEM). Data were analyzed using analysis of variance (ANOVA). Prior to ANOVA, data distributions were visually inspected to assess approximate normality and homogeneity of variance. For the LA and MBB tests, data were analyzed using one-way ANOVA followed by Dunnett’s test. Pairwise comparisons were performed between the control group (5% AG or saline) and each treatment group (single agents) to evaluate the single agent effects, and between the combination group and the other three groups to evaluate the combinatorial effects. For sleep architecture, two-way ANOVA including block and treatment as fixed effects was used. For comparisons involving multiple treatment groups, pairwise comparisons against the vehicle group were performed using Dunnett’s multiple comparisons test. For two-group comparisons, no adjustment for multiplicity was applied. A two-sided *P*-value of less than .05 was considered statistically significant.

## RESULTS

### Combination of Brexpiprazole and Paroxetine in the MBB Test

**Figure 1:**
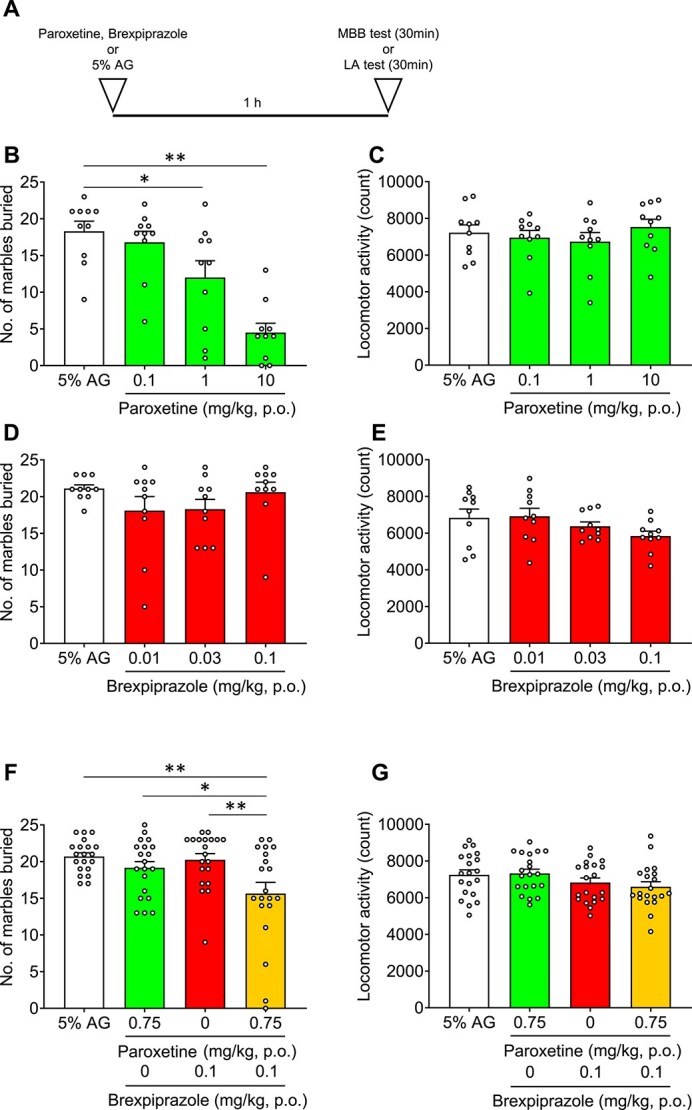
Effects of brexpiprazole, paroxetine, or combination of brexpiprazole and paroxetine on marble-burying behavior and locomotor activity. Each medication or vehicle was administered orally. A: Experimental scheme. B, D, F: Marble-burying behavior test. C, E, G: Locomotor activity test (B, C: Paroxetine only; D, E: Brexpiprazole only; F, G: The combination of brexpiprazole and paroxetine). Data are expressed as mean ± SEM (B-E: *n* = 10; F, G: *n* = 20). Statistical analyses were performed using a one-way using analysis of variance followed by Dunnett’s test. ^*^P < .05, ^**^P < .01 vs 5% AG (A-E), vs combination (F, G). Statistics reported in Supplementary Table 1.

Marble-burying behavior has been variously interpreted as reflecting anxiety-like, compulsive-like, or perseverative behaviors, and MBB test is classically used for screening anti-anxiety effects.^31–34^ We also conducted the LA test to confirm the inhibition of marble-burying behavior without decreasing the lomotor activity. Paroxetine decreased the number of buried marbles in a dose-dependent manner; both 1 and 10 mg/kg paroxetine significantly reduced the number of buried marbles without suppressing LA (Figure 1B, C). Brexpiprazole (0.01-0.1 mg/kg) alone did not affect either the number of buried marbles or LA (Figure 1D, E). We then evaluated the combination of brexpiprazole (0.1 mg/kg) and paroxetine (0.75 mg/kg); these doses had not been found to be effective as single agents in the MBB test. The combination of brexpiprazole and paroxetine resulted in a significantly lower number of buried marbles compared to the vehicle (*P* = .0018), paroxetine alone (*P* = .0417), and brexpiprazole alone groups (*P* = .0049) (Figure 1F). There were no significant changes in LA between the vehicle (*P* = .1827), paroxetine alone (*P* = .1200), and brexpiprazole alone groups (*P* = .8612) (Figure 1G).

### Combination of JP-1302 and Paroxetine in the MBB Test

**Figure 2:**
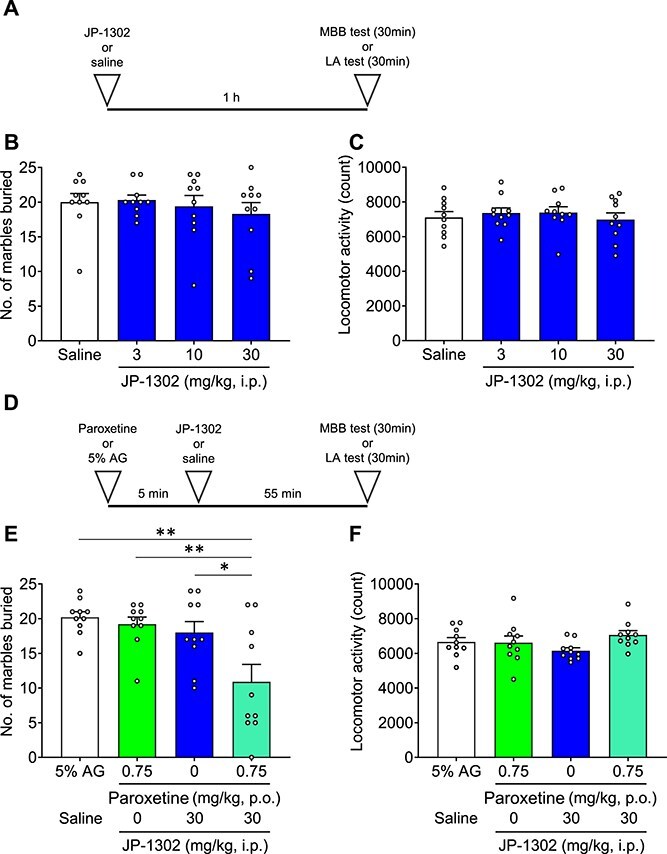
Effects of JP-1302 or combination of JP-1302 and paroxetine on marble-burying behavior and locomotor activity. Each medication, vehicle was administered orally or intraperitoneally. A, D: Experimental scheme. B, E: Marble-burying behavior test. C, F: Locomotor activity test. (B, C: JP-1302 only; E, F: The combination of JP-1302 and paroxetine). Data are expressed as mean ± SEM (*n* = 10). Statistical analyses were performed using a one-way using analysis of variance followed by Dunnett’s test. ^*^P < .05, ^**^P < .01 vs saline (B, C), vs combination (E, F). Statistics reported in Supplementary Table 1.

Brexpiprazole is an antagonist at α_2C_ AR.^8^^,^^35^ It has been reported that JP-1302 (an α_2C_ AR antagonist) suppressed depressive-like behaviors.^36^ Thus, we evaluated JP-1302 in the MBB test (Figure 2A). JP-1302 (3-30 mg/kg) did not affect either the number of buried marbles or LA (Figure 2B, C). We also tested the combination of JP-1302 (30 mg/kg) and paroxetine (0.75 mg/kg), similar to the previous brexpiprazole experiment (Figure 2D). The combination of JP-1302 and paroxetine significantly reduced the number of buried marbles compared to the vehicle (*P* = .0007), paroxetine alone (*P* = .0025), and JP-1302 alone groups (*P* = .0101) (Figure 2E). There were no significant changes in LA among the vehicle (*P* = .5962), paroxetine alone (*P* = .5284), and JP-1302 alone groups (*P* = .0634) (Figure 2F).

### Effects of Brexpiprazole on Sleep Architecture

**Figure 3:**
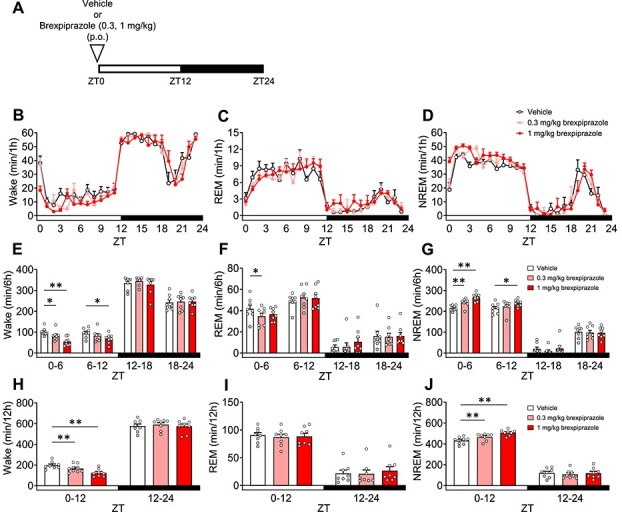
Effects of brexpiprazole on sleep architecture in mice. Each medication or vehicle was administered orally 30 minutes before ZT0. A: Experimental scheme. B-D: Hourly variations in waking, rapid eye movement sleep, and non-rapid eye movement sleep over a 24 h period. E-G: Each 6 h variation in waking, rapid eye movement sleep, and non-rapid eye movement sleep over a 24 h period. H-J: Each 12 h variation in waking, rapid eye movement sleep, and non-rapid eye movement sleep over a 24 h period. Data are expressed as mean ± SEM (*n* = 8). Statistical analyses were performed using a two-way using analysis of variance, including block and treatment as fixed effects, followed by Dunnett’s test in a randomized block design. ^*^P < .05, ^**^P < .01 vs vehicle (E-J). Statistics reported in Supplementary Table 2.

We evaluated the effect of brexpiprazole on sleep architecture. Mice were orally administered 30 minutes before ZT0 (Figure 3A). Both 0.3 and 1 mg/kg brexpiprazole significantly decreased Wake in ZT0-6 and ZT0-12, while 1 mg/kg also significantly reduced Wake in ZT6–12 (Figure 3B, E, H). Additionally, 0.3 mg/kg brexpiprazole significantly decreased REM sleep in ZT0–6 (Figure 3C, F, I). Both doses (0.3 and 1 mg/kg) significantly increased NREM sleep in ZT0–6 and ZT0–12, while 1 mg/kg also led to a significant rise in NREM sleep in ZT6–12 (Figure 3D, G, J). In ZT0–6, brexpiprazole strongly increased NREM sleep. We conducted spectral power analysis in Wake/REM/NREM (Supplementary Figure 1A–C). In NREM sleep, 0.3 and 1 mg/kg brexpiprazole significantly increased delta power in a dose-dependent manner (Supplementary Figure 1D, Supplementary Table 3).

### Effects of Paroxetine on Sleep Architecture

**Figure 4:**
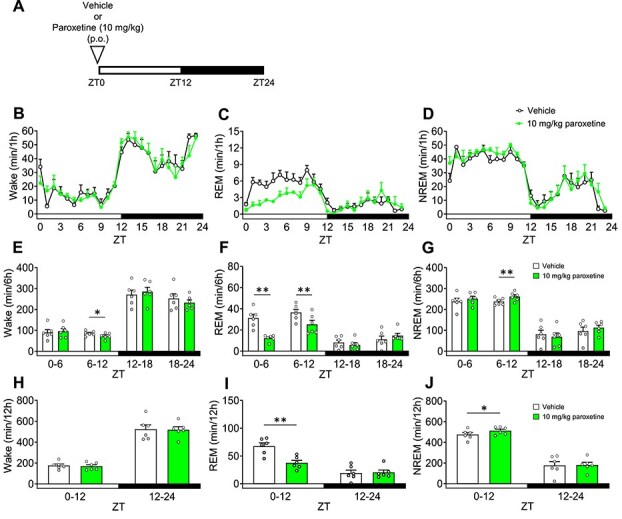
Effects of paroxetine on sleep architecture in mice. Paroxetine or vehicle was administered orally 30 minutes before ZT0. A: Experimental scheme. B-D: Hourly variations in waking, rapid eye movement sleep, and non-rapid eye movement sleep over a 24 h period. E-G: Each 6 h variation in waking, rapid eye movement sleep, and non-rapid eye movement sleep over a 24 h period. H-J: Each 12 h variation in waking, rapid eye movement sleep, and non-rapid eye movement sleep over a 24 h period. Data are expressed as mean ± SEM (*n* = 6). Statistical analyses were performed using a two-way using analysis of variance, including block and treatment as fixed effects in a randomized block design. ^*^P < .05, ^**^P < .01 vs vehicle (E-J). Statistics reported in Supplementary Table 2.

We evaluated the effect of paroxetine (10 mg/kg) on sleep architecture. At this dose, paroxetine produced anti-anxiety effects that were evident in the MBB test (Figure 1B). Mice were administered paroxetine 30 minutes before ZT0 (Figure 4A). Paroxetine significantly decreased Wake in ZT6-12 (Figure 4B, E, H), significantly reduced REM sleep in ZT0-6, ZT6-12, and ZT0-12 (Figure 4C, F, I), and significantly increased NREM sleep in ZT6-12 and ZT0-12 (Figure 4D, G, J). Paroxetine did not affect delta power during NREM sleep (Supplementary Figure 2D, Supplementary Table 3).

### Effects of Combination of Brexpiprazole and Paroxetine on Sleep Architecture

**Figure 5:**
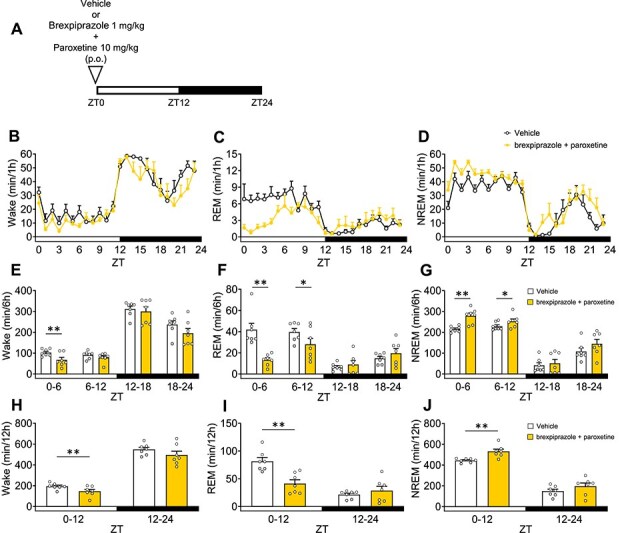
Effects of the combination of brexpiprazole and paroxetine on sleep architecture in mice. Brexpiprazole and paroxetine or vehicle were administered orally 30 minutes before ZT0. A: Experimental scheme. B–D: Hourly variations in waking, rapid eye movement sleep, and non-rapid eye movement sleep over a 24 h period. E–G: Each 6 h variation in waking, rapid eye movement sleep, and non-rapid eye movement sleep over a 24 h period. H–J: Each 12 h variation in waking, REM sleep, and non-rapid eye movement sleep over a 24 h period. Data are expressed as mean ± SEM (*n* = 7). Statistical analyses were performed using a two-way using analysis of variance, including block and treatment as fixed effects in a randomized block design. ^*^P < .05, ^**^P < .01 vs vehicle (E–J). Statistics reported in Supplementary Table 2.

The results mentioned above indicate that brexpiprazole increases NREM sleep, while paroxetine decreases REM sleep. Furthermore, we examined the combined effects of brexpiprazole and paroxetine on sleep architecture. Mice were administered the drugs 30 minutes before ZT0 (Figure 5A). Combined administration significantly decreased Wake in ZT0-6 and ZT0-12 (Figure 5B, E, H), reduced REM sleep in ZT0-6, ZT6-12, and ZT0-12 (Figure 5C, F, I), and increased NREM sleep in ZT0-6, ZT6-12, and ZT0-12 (Figure 5D, G, J). The combination of brexpiprazole and paroxetine also significantly increased delta power during NREM sleep (Supplementary Figure 3D, Supplementary Table 3).

**Figure 6:**
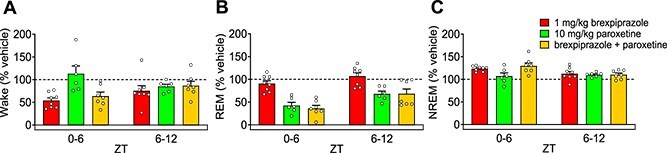
Comparison of the efficacy of brexpiprazole and paroxetine with the combination in ZT0–12. A–C: Each 6 h variation in waking, rapid eye movement sleep, and non-rapid eye movement sleep (%vehicle). Data are expressed as mean ± SEM (brexpiprazole: *n* = 8, paroxetine: *n* = 6, brexpiprazole + paroxetine: *n* = 7).

To compare the efficacy of the combination with each drug, the percentage change in Wake, REM, and NREM stages were calculated based on the results of vehicle administration. Additionally, effects were compared from a pharmacokinetic perspective only during ZT0-12. The reduction in Wake and the increase in NREM sleep were approximately the same as brexpiprazole alone in ZT0-6 (Figure 6A, C). The reduction in REM sleep was similar to that observed with paroxetine alone in ZT0-6 and ZT6-12 (Figure 6B).

## DISCUSSION

In this study, we reported for the first time that the combination of brexpiprazole and paroxetine has positive effects on anxiety-like behavior and sleep architecture in preclinical models. The combination of brexpiprazole and paroxetine (each at doses not effective as single agents) produced anti-anxiety effects (Figure 1). Similarly, the combination of JP-1302 (an α_2C_ AR antagonist) and paroxetine also demonstrated anti-anxiety effects (Figure 2). These results were not associated with a decrease in lomotor activity. Regarding sleep architecture, brexpiprazole reduced Wake and increased NREM sleep. During ZT0-6, REM sleep was also reduced, but this effect was approximately 15% and did not show a dose–response relationship, suggesting that the reduction in REM sleep was secondary to the increase in NREM sleep (Figure 3). Moreover, brexpiprazole increased delta power during NREM sleep (Supplementary Figure 1). Paroxetine decreased REM sleep. During ZT6-12, NREM sleep was increased (Figure 4). The combined administration of brexpiprazole and paroxetine resulted in reduced Wake and REM sleep, and increased NREM sleep (Figure 5), and increased delta power during NREM sleep (Supplementary Figure 3). To compare the efficacy of the combination therapy with each drug alone, the reduction in Wake and the increase in NREM sleep were approximately the same as brexpiprazole alone, while the reduction in REM sleep was similar to that observed with paroxetine alone. These results suggest that the combination may have effects on sleep architecture without interfering with the mechanisms of each drug (Figure 6).

Patients with MDD often exhibit anxiety symptoms, and MDD with anxiety tends to be more severe and less responsive to antidepressants, reducing quality of life.^37^^,^^38^ Additionally, it has been reported that MDD patients treated with antidepressants exhibit anxiety as a residual symptom.^39^ In this study, we evaluated anti-anxiety effects with the MBB test. The MBB test utilizes the behavioral trait of rodents to bury harmless glittering objects. This behavior has been variably interpreted as reflecting is classically considered to be a model of anxiety-like, compulsive-like, or perseverative behaviors, and the MBB test is commonly used to evaluate the level of anxiety.^31–34^

We were able to demonstrate that the combination of brexpiprazole and paroxetine produced anti-anxiety effects (Figure 1). These results are consistent with the report that adjunctive brexpiprazole with an SSRI improved both the Obsession and Compulsion subscales of the Y-BOCS in patients with SSRI-resistant obsessive-compulsive disorder, as well as the report that adjunctive brexpiprazole improved the HAM-A score in patients with MDD and anxiety symptoms.^40^^,^^41^ Importantly, brexpiprazole has minimal inhibitory effects on CYP2D6 and therefore is not expected to increase paroxetine blood levels.^42^ We hypothesized that the α_2C_ AR antagonistic activity of brexpiprazole is related to this combination effect. The α_2C_ AR is a subtype of the α_2_ AR, expressed in the hippocampus, striatum, and frontal cortex, which are stress-regulatory regions. The α_2C_ AR negatively regulates noradrenaline and serotonin release at presynapses as an autoreceptor or heteroreceptor, respectively.^43–46^ In addition, it has been reported that α_2_ AR is increased in the hippocampus and frontal cortex of depressed suicide victims, and α_2_ AR upregulation can be normalized with antidepressant treatment.^47^^,^^48^ Therefore, many studies have been conducted to investigate the relationship between α_2C_ AR and MDD. For example, JP-1302 (an α_2C_ AR antagonist) decreased immobility time in the FST.^36^ Moreover, in the Flinders Sensitive Line rat, characterized by depression-like behavior, ORM-10921 (an α_2C_ AR antagonist) decreased immobility time in the FST, and improved the reduction in serotonin levels and abnormalities in serotonin metabolism in the hippocampus.^49^^,^^50^ We discovered that the combination of JP-1302 and paroxetine reduced anxiety-like behavior (Figure 2). This result suggests that the 5-HT and noradrenaline upregulation via α_2C_ AR antagonistic activity enhanced SSRI effects. Therefore, the anti-anxiety effect of combining brexpiprazole and paroxetine may also be attributed to the α_2C_ AR antagonistic activity of brexpiprazole. On the other hand, the 5-HT_1A_ receptor partial agonistic activity and 5-HT_2A_ receptor antagonistic activity of brexpiprazole could also make a contribution because tandospirone (a 5-HT_1A_ receptor agonist) produced an anti-anxiety effect in the MBB test, and pimavanserin (a 5-HT_2A_ receptor inverse agonist) reversed anxiety-like behavior in PTSD model rats.^51^^,^^52^

MDD patients exhibit various sleep disturbances, such as lower sleep efficiency, longer REM sleep duration, and diminished slow-wave activity (SWA) during NREM sleep.^53^ About two-thirds of patients with MDD fail to achieve remission even after trialing multiple treatments, and sleep disturbance has often been observed as a residual symptom.^6^ It has been reported that adjunctive brexpiprazole for MDD patients treated with antidepressants improved not only depression scores (Montgomery–Åsberg Depression Rating Scale) but also circadian rhythm scores (Biological Rhythms Interview of Assessment in Neuropsychiatry).^20^^,^^21^ In this study, we recorded an EEG from the cortex and an EMG from the trapezius and evaluated the effects on sleep architecture. Furthermore, to ensure a robust circadian rhythm, we conducted this experiment with a running wheel.

Brexpiprazole dose-dependently decreased Wake and increased NREM sleep (Figure 3). In NREM sleep, brexpiprazole increased delta power in a dose-dependent manner (Supplementary Figure 1). SWA and delta waves are closely related concepts in the context of sleep, and SWA is also a reliable indicator of sleep intensity or sleep depth, with high levels during deep NREM sleep.^54^ These results were consistent with the previous report that adding brexpiprazole for patients with schizophrenia on haloperidol treatment increased N2 and N3 stages of NREM sleep.^55^ Due to the similar binding affinities (Ki values) for D_2_ and 5-HT_2A_ receptors, it is expected to exhibit activity at the same dose.^8^ D_2_ receptor knock-out mice exhibited a decrease in Wake and increases in REM sleep and NREM sleep during the light phase.^56^ It has been reported MDL100907 (a 5-HT_2A_ receptor antagonist) increase NREM sleep and delta power during NREM sleep in rodent, SR46349 (a 5-HT_2A_ receptor antagonist) increased SWA in healthy volunteers.^57^^,^^58^ In a clinical study, 2nd generation antipsychotics (olanzapine, quetiapine, ziprasidone), which have D_2_ receptor antagonistic activity and 5-HT_2A_ receptor antagonistic activity, increased total sleep time and sleep efficacy in healthy volunteers.^59^^,^^60^ These reports suggest that brexpiprazole increased NREM sleep via D_2_ receptor partial agonistic activity and 5-HT_2A_ receptor antagonistic activity, which might increase delta power. Moreover, in a depressive rat model induced with adrenocorticotropic hormone, tandospirone (a 5-HT_1A_ receptor partial agonist) decreased Wake and increased NREM sleep, suggesting that the 5-HT_1A_ receptor partial agonistic activity of brexpiprazole may be involved in sleep architecture changes.^61^ The patients with MDD exhibited a decrease in REM latency and an increase in REM duration.^53^ Both in MDD patients and healthy volunteers, SSRI antidepressants increased REM latency and decreased total REM sleep.^62–64^ In this study, we were able to show that paroxetine also decreased REM sleep in mice (Figure 4). The combination of brexpiprazole and paroxetine decreased Wake and REM sleep and increased NREM sleep (Figure 5). Moreover, the treatment increased delta power during NREM sleep (Supplementary Figure 3). Comparing the efficacy of each drug when administered alone and in combination, the effects of decrease in Wake and increase in NREM sleep were approximately the same as brexpiprazole alone, and the effect of decrease in REM sleep was approximately the same as paroxetine alone (Figure 6). In patients with MDD, not only was REM sleep increased, but the amount of NREM sleep was decreased.^53^ Adjunctive brexpiprazole may improve the pathology of MDD by increasing NREM sleep to address the abnormal sleep architecture that remains with SSRI monotherapy.

In this non-clinical study, we clarified the anti-anxiety effects and the effects on sleep architecture of combination therapy with brexpiprazole and paroxetine in mice. Analysis results from a randomized double-blind placebo-controlled trial reported that adjunctive therapy with brexpiprazole improved core depressive symptoms and sleep disturbances in patients experiencing MDD and anxious distress whose response to antidepressants had been inadequate.^65^ In previous studies, the combination of brexpiprazole and antidepressants improved depressive-like behavior mediated by the BDNF–TrkB pathway.^16^^,^^17^ Other pathways were not investigated. Our work suggests that in adjunctive brexpiprazole with an SSRI, α_2C_ AR antagonistic activity is involved in anti-anxiety effects; additionally, D_2_ receptor partial agonistic activity and 5-HT_2A_ receptor antagonistic activity may contribute to the increase in NREM sleep. These findings suggest that the combination of brexpiprazole and paroxetine may have potential to address residual symptoms in MDD, particularly anxiety and sleep disturbances, although further clinical validation is required.

It is important to note several limitations regarding the translational relevance of our findings. All experiments in this study were conducted in naïve mice, whereas the neurobiological state in MDD patients differs substantially. Stress-based depression models in animals, such as social defeat and restraint stress, show both behavioral abnormalities and alterations in sleep architecture, including anxiety-like behaviors and increased NREM and REM sleep.^66^^,^^67^ Moreover, it has been shown that repeated social defeat stress reduces stress-induced dopaminergic responses in the mPFC.^68^^,^^69^ Therefore, future studies using stress-exposed or depression-relevant animal models are needed to more accurately evaluate the effects of brexpiprazole and paroxetine combination under conditions that more closely mimic patients with MDD. Furthermore, it should be noted that increases in NREM sleep and delta power do not necessarily imply an improvement in sleep quality. Therefore, further studies are needed to differentiate restorative sleep from pharmacologically induced sleep states.

## Supplementary Material

Supplementary_materials_pyag007

## Data Availability

The authors declare that all data supporting the findings of this study are contained within the paper.
