# Supplementary material for: Effects of adjunctive brexpiprazole with selective serotonin reuptake inhibitor treatment on anxiety and sleep architecture in mice
Source: Int J Neuropsychopharmacol. 2026 Feb 20;29(4):pyag007. doi: 10.1093/ijnp/pyag007 (PMC13070398; doi:10.1093/ijnp/pyag007)
Supplement: Supplementary_materials_pyag007 [file supplementary_materials_pyag007.docx]

**Effects of Adjunctive Brexpiprazole with SSRI Treatment on Anxiety and Sleep Architecture in Mice**

**Junya Maruoka, Kohei Kozuka, Ryo Egami, Yusuke Kubo, Yusuke Kakumoto, Tetsuro Kikuchi and Kazuhiko Kume.**

**This Supplementary Data file includes:**

**Supplementary Figure 1 to 3**

**Supplementary Tables 1 to 3**

**A**

**B**

**C**

**

**

**D**

**Supplementary Figure 1. Effects of brexpiprazole on normalized power in ZT0–6.**

Each medication or vehicle was administered orally 30 minutes before ZT0. A–C: Normalized power of mice during waking, REM sleep and NREM sleep. D: The AUC of the delta wave (0.75–4 Hz) of the normalized power was measured during NREM sleep. Data are expressed as mean ± SEM (N=8). Statistical analyses were performed using a two-way ANOVA including block and treatment as fixed effects followed by Dunnett’s test in a randomized block design. *p<0.05, **p<0.01 vs. Vehicle (D). Statistics reported in Supplementary Table 3.

**A**

**B**

**C**

**D**

**Supplementary Figure 2. Effects of paroxetine on normalized power in ZT0–6.**

Each medication or vehicle was administered orally 30 minutes before ZT0. A–C: Normalized power of mice during waking, REM sleep and NREM sleep. D: The AUC of the delta wave of the normalized power was measured during NREM sleep. Data are expressed as mean ± SEM (N=6). Statistical analyses were performed using a two-way ANOVA including block and treatment as fixed effects followed in a randomized block design. *p<0.05, **p<0.01 vs. Vehicle (D). Statistics reported in Supplementary Table 3.

**

**A**

**B**

**C**

**D**

**Supplementary Figure 3. Effects of the combination of brexpiprazole and paroxetine on normalized power in ZT0–6.**

Brexpiprazole and paroxetine or vehicle was administered orally 30 minutes before ZT0. A–C: Normalized power of mice during waking, REM sleep and NREM sleep. D: The AUC of the delta wave of the normalized power was measured during NREM sleep. Data are expressed as mean ± SEM (N=7). Statistical analyses were performed using a two-way ANOVA including block and treatment as fixed effects followed in a randomized block design. *p<0.05, **p<0.01 vs. Vehicle (D). Statistics reported in Supplementary Table 3.

|  | **Group** | | **MBB test**  #  * | | | | | | **LA test** | | | | | |
| --- | --- | --- | --- | --- | --- | --- | --- | --- | --- | --- | --- | --- | --- | --- |
|  |  |  | **N** | **Burried marbles** | | | **P value** | **P value combination** | **N** | **Locomotor activity** | | | **P value**  * | **P value combination**  # |
|  |  |  |  | **Mean** | **±** | **SE** |  |  |  | **Mean** | **±** | **SE** |  |  |
| **Paroxetine** |  | **5% AG** | **10** | **18.3** | **±** | **1.4** | **-** |  | **10** | **7221** | **±** | **425.0** | **-** |  |
|  | **0.1 mg/kg** | **paroxetine** | **10** | **16.8** | **±** | **1.5** | **0.8580** |  | **10** | **6947** | **±** | **394.4** | **0.9453** |  |
|  | **1 mg/kg** | **paroxetine** | **10** | **12.0** | **±** | **2.3** | **0.0284** |  | **10** | **6729** | **±** | **499.1** | **0.7668** |  |
|  | **10 mg/kg** | **paroxetine** | **10** | **4.5** | **±** | **1.3** | **<0.0001** |  | **10** | **7528** | **±** | **426.2** | **0.9260** |  |
| **Brexpiprazole** |  | **5% AG** | **10** | **21.1** | **±** | **0.5** | **-** |  | **10** | **6836** | **±** | **473.5** | **-** |  |
|  | **0.01 mg/kg** | **brexpiprazole** | **10** | **18.1** | **±** | **1.9** | **0.2983** |  | **10** | **6915** | **±** | **439.8** | **0.9975** |  |
|  | **0.03 mg/kg** | **brexpiprazole** | **10** | **18.3** | **±** | **1.3** | **0.3511** |  | **10** | **6375** | **±** | **233.9** | **0.7084** |  |
|  | **0.1 mg/kg** | **brexpiprazole** | **10** | **20.6** | **±** | **1.4** | **0.9882** |  | **10** | **5840** | **±** | **267.7** | **0.1559** |  |
| **Brexpiprazole + Paroxetine** |  | **5% AG** | **20** | **20.7** | **±** | **0.5** |  | **0.0018** | **20** | **7247** | **±** | **269.4** |  | **0.1827** |
|  | **0.75 mg/kg** | **paroxetine** | **20** | **19.2** | **±** | **0.9** |  | **0.0417** | **20** | **7322** | **±** | **233.4** |  | **0.1200** |
|  | **0.1 mg/kg** | **brexpiprazole** | **20** | **20.3** | **±** | **0.8** |  | **0.0049** | **20** | **6829** | **±** | **243.4** |  | **0.8612** |
|  | **brexpiprazole + paroxetine** | | **20** | **15.7** | **±** | **1.5** |  | **-** | **20** | **6600** | **±** | **268.6** |  | **-** |
| **JP-1302** |  | **saline** | **10** | **20.0** | **±** | **1.2** | **-** |  | **10** | **7107** | **±** | **337.0** | **-** |  |
|  | **3 mg/kg** | **JP-1302** | **10** | **20.3** | **±** | **0.7** | **0.9972** |  | **10** | **7359** | **±** | **297.8** | **0.9152** |  |
|  | **10 mg/kg** | **JP-1302** | **10** | **19.4** | **±** | **1.6** | **0.9785** |  | **10** | **7387** | **±** | **334.1** | **0.8888** |  |
|  | **30 mg/kg** | **JP-1302** | **10** | **18.3** | **±** | **1.6** | **0.6995** |  | **10** | **6980** | **±** | **386.1** | **0.9873** |  |
| **JP-1302 + Paroxetine** | **5% AG + saline** | | **10** | **20.2** | **±** | **0.8** |  | **0.0007** | **10** | **6656** | **±** | **258.0** |  | **0.5962** |
|  | **0.75 mg/kg** | **paroxetine** | **10** | **19.2** | **±** | **1.0** |  | **0.0025** | **10** | **6616** | **±** | **385.4** |  | **0.5284** |
|  | **30 mg/kg** | **JP-1302** | **10** | **18.0** | **±** | **1.6** |  | **0.0101** | **10** | **6149** | **±** | **172.8** |  | **0.0634** |
|  | **JP-1302 + paroxetine** | | **10** | **10.9** | **±** | **2.5** |  | **-** | **10** | **7065** | **±** | **244.3** |  | **-** |

**Supplementary Table 1. Statistics for the MBB test and the LA test.**

Brexpiprazole, paroxetine and 5% AG were orally administered, JP-1302 and saline were administered intraperitoneally. Statistical analyses were performed using a one-way ANOVA followed by Dunnett’s test. (*: vs. 5% AG or saline.) (#: vs. combination.)

|  |  |  | **Group** | | **Time (min)** | | | **P value** |  | **Group** | | **Time (min)** | | | **P value** |
| --- | --- | --- | --- | --- | --- | --- | --- | --- | --- | --- | --- | --- | --- | --- | --- |
|  |  |  |  |  | **Mean** | **±** | **SE** |  |  |  |  | **Mean** | **±** | **SE** |  |
| **Brexpiprazole (N=8)**  * | **Wake** | **ZT0–6** | **Vehicle** | | **101.3** | **±** | **6.3** | **_** | **ZT0–12** |  | **Vehicle** | **197.4** | **±** | **12.8** | **_** |
|  |  |  | **0.3 mg/kg** | **brexpiprazole** | **82.8** | **±** | **8.3** | **0.0379** |  | **0.3 mg/kg** | **brexpiprazole** | **166.4** | **±** | **13.8** | **0.0024** |
|  |  |  | **1 mg/kg** | **brexpiprazole** | **55.1** | **±** | **7.3** | **<0.0001** |  | **1 mg/kg** | **brexpiprazole** | **124.0** | **±** | **13.0** | **<0.0001** |
|  |  | **ZT6–12** | **Vehicle** | | **96.1** | **±** | **8.1** | **_** |  |  |  |  |  |  |  |
|  |  |  | **0.3 mg/kg** | **brexpiprazole** | **83.6** | **±** | **9.4** | **0.2660** |  |  |  |  |  |  |  |
|  |  |  | **1 mg/kg** | **brexpiprazole** | **68.8** | **±** | **8.0** | **0.0108** |  |  |  |  |  |  |  |
|  | **REM** | **ZT0–6** | **Vehicle** | | **41.7** | **±** | **3.5** | **_** | **ZT0–12** |  | **Vehicle** | **90.6** | **±** | **4.7** | **_** |
|  |  |  | **0.3 mg/kg** | **brexpiprazole** | **34.7** | **±** | **2.9** | **0.0251** |  | **0.3 mg/kg** | **brexpiprazole** | **87.0** | **±** | **5.4** | **0.6833** |
|  |  |  | **1 mg/kg** | **brexpiprazole** | **36.6** | **±** | **2.1** | **0.1046** |  | **1 mg/kg** | **brexpiprazole** | **88.4** | **±** | **5.5** | **0.8679** |
|  |  | **ZT6–12** | **Vehicle** | | **48.9** | **±** | **2.4** | **_** |  |  |  |  |  |  |  |
|  |  |  | **0.3 mg/kg** | **brexpiprazole** | **52.3** | **±** | **3.5** | **0.5877** |  |  |  |  |  |  |  |
|  |  |  | **1 mg/kg** | **brexpiprazole** | **51.8** | **±** | **3.6** | **0.6672** |  |  |  |  |  |  |  |
|  | **NREM** | **ZT0–6** | **Vehicle** | | **217.0** | **±** | **4.7** | **_** | **ZT0–12** |  | **Vehicle** | **432.0** | **±** | **12.4** | **_** |
|  |  |  | **0.3 mg/kg** | **brexpiprazole** | **242.5** | **±** | **6.9** | **0.0023** |  | **0.3 mg/kg** | **brexpiprazole** | **466.6** | **±** | **11.9** | **0.0007** |
|  |  |  | **1 mg/kg** | **brexpiprazole** | **268.2** | **±** | **6.6** | **<0.0001** |  | **1 mg/kg** | **brexpiprazole** | **507.6** | **±** | **10.0** | **<0.0001** |
|  |  | **ZT6–12** | **Vehicle** | | **215.0** | **±** | **8.7** | **_** |  |  |  |  |  |  |  |
|  |  |  | **0.3 mg/kg** | **brexpiprazole** | **224.1** | **±** | **10.2** | **0.4931** |  |  |  |  |  |  |  |
|  |  |  | **1 mg/kg** | **brexpiprazole** | **239.4** | **±** | **5.9** | **0.0260** |  |  |  |  |  |  |  |
| **Paroxetine (N=6)** | **Wake** | **ZT0–6** | **Vehicle** | | **90.6** | **±** | **13.4** | **-** | **ZT0–12** |  | **Vehicle** | **176.4** | **±** | **14.4** | **-** |
|  |  |  | **10 mg/kg** | **paroxetine** | **96.2** | **±** | **12.5** | **0.7178** |  | **10 mg/kg** | **paroxetine** | **169.2** | **±** | **14.3** | **0.6624** |
|  |  | **ZT6–12** | **Vehicle** | | **85.9** | **±** | **4.8** | **-** |  |  |  |  |  |  |  |
|  |  |  | **10 mg/kg** | **paroxetine** | **73.0** | **±** | **5.6** | **0.0360** |  |  |  |  |  |  |  |
|  | **REM** | **ZT0–6** | **Vehicle** | | **31.3** | **±** | **3.5** | **-** | **ZT0–12** |  | **Vehicle** | **67.8** | **±** | **5.9** | **-** |
|  |  |  | **10 mg/kg** | **paroxetine** | **12.4** | **±** | **1.2** | **0.0039** |  | **10 mg/kg** | **paroxetine** | **37.6** | **±** | **4.5** | **0.0011** |
|  |  | **ZT6–12** | **Vehicle** | | **36.6** | **±** | **3.0** | **-** |  |  |  |  |  |  |  |
|  |  |  | **10 mg/kg** | **paroxetine** | **25.3** | **±** | **3.8** | **0.0035** |  |  |  |  |  |  |  |
|  | **NREM** | **ZT0–6** | **Vehicle** | | **238.2** | **±** | **14.9** | **-** | **ZT0–12** |  | **Vehicle** | **475.8** | **±** | **19.4** | **-** |
|  |  |  | **10 mg/kg** | **paroxetine** | **251.4** | **±** | **11.6** | **0.4051** |  | **10 mg/kg** | **paroxetine** | **513.2** | **±** | **14.7** | **0.0487** |
|  |  | **ZT6–12** | **Vehicle** | | **237.6** | **±** | **6.9** | **-** |  |  |  |  |  |  |  |
|  |  |  | **10 mg/kg** | **paroxetine** | **261.7** | **±** | **8.5** | **0.0034** |  |  |  |  |  |  |  |
| **Brexpiprazole + Paroxetine (N=7)** | **Wake** | **ZT0–6** | **Vehicle** | | **102.9** | **±** | **6.4** | **-** | **ZT0–12** |  | **Vehicle** | **194.4** | **±** | **11.1** | **-** |
|  |  |  | **brexpiprazole + paroxetine** | | **67.8** | **±** | **12.1** | **0.0050** |  | **brexpiprazole + paroxetine** | | **147.0** | **±** | **18.2** | **0.0015** |
|  |  | **ZT6–12** | **Vehicle** | | **91.4** | **±** | **6.8** | **-** |  |  |  |  |  |  |  |
|  |  |  | **brexpiprazole + paroxetine** | | **79.2** | **±** | **8.5** | **0.1465** |  |  |  |  |  |  |  |
|  | **REM** | **ZT0–6** | **Vehicle** | | **41.9** | **±** | **6.0** | **-** | **ZT0–12** |  | **Vehicle** | **81.5** | **±** | **6.9** | **-** |
|  |  |  | **brexpiprazole + paroxetine** | | **13.3** | **±** | **2.0** | **0.0088** |  | **brexpiprazole + paroxetine** | | **41.4** | **±** | **6.9** | **0.0064** |
|  |  | **ZT6–12** | **Vehicle** | | **39.6** | **±** | **3.3** | **-** |  |  |  |  |  |  |  |
|  |  |  | **brexpiprazole + paroxetine** | | **28.1** | **±** | **5.5** | **0.0242** |  |  |  |  |  |  |  |
|  | **NREM** | **ZT0–6** | **Vehicle** | | **215.2** | **±** | **5.5** | **-** | **ZT0–12** |  | **Vehicle** | **444.1** | **±** | **7.5** | **-** |
|  |  |  | **brexpiprazole + paroxetine** | | **278.9** | **±** | **13.6** | **0.0029** |  | **brexpiprazole + paroxetine** | | **531.6** | **±** | **21.7** | **0.0024** |
|  |  | **ZT6–12** | **Vehicle** | | **228.9** | **±** | **7.1** | **-** |  |  |  |  |  |  |  |
|  |  |  | **brexpiprazole + paroxetine** | | **252.7** | **±** | **11.4** | **0.0328** |  |  |  |  |  |  |  |

**Supplementary Table 2. Statistics for sleep architecture in ZT0–12.**

Brexpiprazole, paroxetine and vehicle were orally administered. Statistical analyses were performed using a two-way ANOVA including block and treatment as fixed effects in a randomized block design. (*: using a two-way ANOVA including block and treatment as fixed effects followed by Dunnett’s test) vs. Vehicle.

| **NREM delta power** | **Group** | | **Mean** | **±** | **SE** | **P value** |
| --- | --- | --- | --- | --- | --- | --- |
| **Brexpiprazole (N=8)**  * | **Vehicle** | | **7.10** | **±** | **0.37** | **_** |
|  | **0.3 mg/kg** | **brexpiprazole** | **7.65** | **±** | **0.34** | **0.0011** |
|  | **1 mg/kg** | **brexpiprazole** | **7.98** | **±** | **0.38** | **<0.0001** |
| **Paroxetine (N=6)** | **Vehicle** | | **8.53** | **±** | **0.58** | **_** |
|  | **10 mg/kg** | **paroxetine** | **8.59** | **±** | **0.57** | **0.5582** |
| **Brexpiprazole + Paroxetine (N=7)** | **Vehicle** | | **6.50** | **±** | **0.24** | **_** |
|  | **brexpiprazole + paroxetine** | | **7.22** | **±** | **0.29** | **0.0041** |

**Supplementary Table 3. Statistics for NREM delta power in ZT0–6.**

Brexpiprazole, paroxetine and vehicle were orally administered. Statistical analyses were performed using a two-way ANOVA including block and treatment as fixed effects in a randomized block design. (*: using a two-way ANOVA including block and treatment as fixed effects followed by Dunnett’s test) vs. Vehicle.
